# Complementary subicular pathways to the anterior thalamic nuclei and mammillary bodies in the rat and macaque monkey brain

**DOI:** 10.1111/ejn.13208

**Published:** 2016-03-06

**Authors:** Kat Christiansen, Christopher M. Dillingham, Nicholas F. Wright, Richard C. Saunders, Seralynne D. Vann, John P. Aggleton

**Affiliations:** ^1^ School of PsychologyCardiff UniversityTower Building 70, Park PlaceCardiffCF10 3ATUK; ^2^ Laboratory of NeuropsychologyNational Institute of Mental HealthBethesdaMDUSA

**Keywords:** anatomy, fornix, hippocampus, memory, subiculum

## Abstract

The origins of the hippocampal (subicular) projections to the anterior thalamic nuclei and mammillary bodies were compared in rats and macaque monkeys using retrograde tracers. These projections form core components of the Papez circuit, which is vital for normal memory. The study revealed a complex pattern of subicular efferents, consistent with the presence of different, parallel information streams, whose segregation appears more marked in the rat brain. In both species, the cells projecting to the mammillary bodies and anterior thalamic nuclei showed laminar separation but also differed along other hippocampal axes. In the rat, these diencephalic inputs showed complementary topographies in the proximal–distal (columnar) plane, consistent with differential involvement in object‐based (proximal subiculum) and context‐based (distal subiculum) information. The medial mammillary inputs, which arose along the anterior–posterior extent of the rat subiculum, favoured the central subiculum (septal hippocampus) and the more proximal subiculum (temporal hippocampus). In contrast, anterior thalamic inputs were largely confined to the dorsal (i.e. septal and intermediate) subiculum, where projections to the anteromedial nucleus favoured the proximal subiculum while those to the anteroventral nucleus predominantly arose in the distal subiculum. In the macaque, the corresponding diencephalic inputs were again distinguished by anterior–posterior topographies, as subicular inputs to the medial mammillary bodies predominantly arose from the posterior hippocampus while subicular inputs to the anteromedial thalamic nucleus predominantly arose from the anterior hippocampus. Unlike the rat, there was no clear evidence of proximal–distal separation as all of these medial diencephalic projections preferentially arose from the more distal subiculum.

## Introduction

Analyses of different functions within the hippocampus often involve comparisons among its principal subareas, i.e. dentate gyrus, CA fields and subiculum (Lee *et al*., 2005; O'Mara, 2005; Hunsaker *et al*., 2008; Chinnakkaruppan *et al*., 2014; Hitti & Siegelbaum, 2014; Schlighting *et al*., 2014) or comparisons along its anterior–posterior (AP) axis (Moser *et al*., 1995; Colombo *et al*., 1998; Bannerman *et al*., 1999; Fanselow & Dong, 2010; Poppenck & Moscovitch, 2011; Aggleton, 2012; Poppenck *et al*., 2013; Strange *et al*., 2014; Chase *et al*., 2015). [Note that in the rodent, the temporal (ventral) hippocampus is homologous with the primate anterior hippocampus, while the septal (dorsal) hippocampus is homologous with the primate posterior hippocampus (Strange *et al*., 2014)]. A further distinction also exists between the ‘proximal’ and ‘distal’ portion of each hippocampal subarea. The presence of specific intrinsic and extrinsic connection patterns that reflect their proximal–distal locations (Naber *et al*., 2000; Witter, 2006; van Strien *et al*., 2009) provides new perspectives on how hippocampal functions might be segregated (Henriksen *et al*., 2010; Kim *et al*., 2012; Nakamura *et al*., 2013).

The present study mapped the topographic organisation of the hippocampal neurons projecting to the anterior thalamic nuclei and mammillary bodies (MBs) in the rat (Experiment 1) and monkey (Experiment 2). These projections arise from the subiculum (Meibach & Siegel, 1977; Rosene & Van Hoesen, 1977; Sikes *et al*., 1977; Swanson & Cowan, 1977; Aggleton *et al*., 1986, 2005), in which the ‘proximal’ subiculum is closest to the CA1 border while the ‘distal’ subiculum is furthest from CA1, i.e. adjacent to the presubiculum. These diencephalic connections, which are core elements of the Papez circuit, have been repeatedly implicated in human episodic memory as well as proving vital for rodent spatial memory (Delay & Brion, 1969; Sutherland & Rodriguez, 1989; Byatt & Dalrymple‐Alford, 1996; Aggleton & Saunders, 1997; Harding *et al*., 2000; Tsivilis *et al*., 2008; Carlesimo *et al*., 2011), thereby creating an ‘extended hippocampal system’ for learning and memory (Aggleton & Brown, 1999; Carlesimo *et al*., 2015). While previous tracer studies have described these connections in both rats (e.g. Meibach & Siegel, 1977; Naber & Witter, 1998; Ishizuka, 2001; Wright *et al*., 2013) and monkeys (Krayniak *et al*., 1979; Aggleton *et al*., 1986, 2005; Xiao & Barbas, 2002b; Saunders *et al*., 2005), there has been little attempt to quantify or compare the sources of these projections across species.

To address these issues, rats (Experiment 1) and macaque monkeys (Experiment 2) were examined after injections of retrograde tracers involving either the medial mammillary nucleus or the anteromedial and anteroventral thalamic nuclei (AM and AV respectively). One goal was to ascertain the potential for parallel, high‐resolution information streams to the medial diencephalon (Aggleton *et al*., 2010; Jankowski *et al*., 2015). The study did not investigate inputs to the anterodorsal thalamic nucleus or the lateral mammillary nucleus as both comprise part of the head‐direction system (Taube, 2007), which has very different patterns of hippocampal inputs that largely involve the postsubiculum and presubiculum (van Groen & Wyss, 1990a,b; Hopkins, 2005; Yoder & Taube, 2011).

## Materials and methods

### Experiment 1: rats

#### Anatomical nomenclature

The flexure of the rat hippocampus makes the terms ‘anterior’ and ‘posterior’ potentially misleading. As already noted, the rat temporal (or ventral) hippocampus corresponds to the primate anterior hippocampus, while the septal (or dorsal) hippocampus corresponds to the posterior hippocampus (Strange *et al*., 2014). To maintain consistency with primate studies, the temporal–septal plane of the rat hippocampus is described as AP.

**Figure 1:**
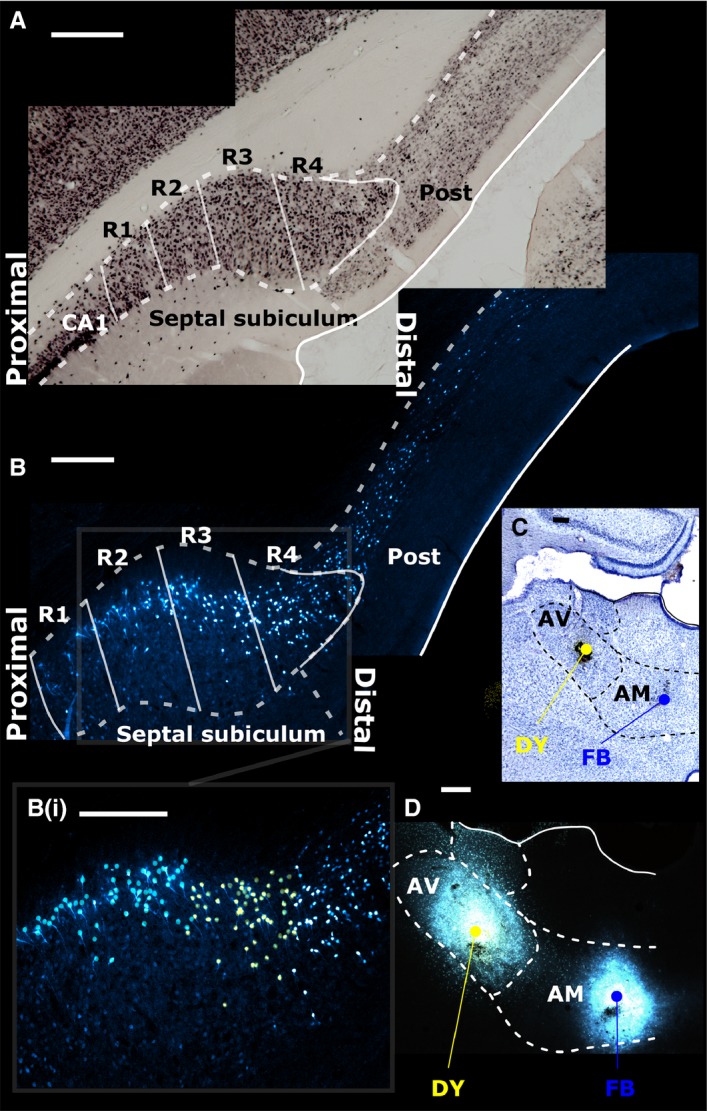
(A) The rat septal (dorsal) subiculum was divided into four regions of interest (R1–4) with R1 most proximal to the CA1 subfield of the hippocampus and R4 most distal. The top photomicrograph (A) is a coronal section from the left hemisphere (NeuN immunostaining). (C and D) Following neuronal tracer injections into the AV of DY and into the AM of FB, labelled cells in each subicular region were visualised (case 45_14). (B) The AV injection labelled relatively more neurons in the distal subiculum, extending into the postsubiculum (Post; marked in yellow in inset B_(i)_). Conversely, the AM injection labelled more proximally situated neurons (marked blue in inset B_(i)_). Scale bars, 250 μm.

Unless specified, the rat anatomical designations follow Swanson (1992). The major division within the MBs consists of the lateral and medial nuclei, with the latter further subdivided in the rat into pars lateralis, pars medialis, pars basalis, pars posterior and pars medianus (Allen & Hopkins, 1988; Vann, 2010). Of these divisions, pars medialis and pars lateralis are the largest. The AM, AV and anterodorsal thalamic nuclei make up the principal anterior thalamic nuclei. In the rat, unlike in the monkey, there is an additional midline area, the interanteromedial nucleus (Swanson, 1992). The limits of the rat subiculum (Fig. 1), presubiculum, parasubiculum and postsubiculum follow Swanson *et al*. (1987). The laminae descriptions for the subiculum match Kloosterman *et al*. (2003), so that the subiculum consists of a superficial molecular layer and a deeper, thick layer of pyramidal cells. The term ‘intermediate subiculum’ (Bast, 2007; Strange *et al*., 2014) refers to the region of the subiculum where the septal subiculum and temporal subiculum converge, i.e. at the flexure of the hippocampus.

#### Subjects

Seventeen male Lister Hooded (LH) rats and two male Dark Agouti (DA) rats (LH weighing 270–300 g and DA weighing 180–220 g; Harlan, Bicester, UK) were used for the tracer analyses. Twelve of these cases had been previously used to describe other anatomical properties of the MBs (*n* = 6) and anterior thalamic nuclei (*n* = 6; see Wright *et al*., 2010, 2013). Five new cases with MB tracer injections were included.

In three additional male LH rats (Harlan, Bicester, UK; 220–270 g), coronal sections were stained for neuronal nuclear antigen (NeuN; Jongên‐Relo & Feldon, 2002), which is not present in glial cells. These cases were used to count neuron numbers within different proximal–distal divisions of the subiculum. All experiments with rats were in accordance with the UK Animals (Scientific Procedures Act, 1986) and associated guidelines, as well as EU directive 2010/63/EU. The study was also approved by local ethical review committees at Cardiff University.

#### Surgical methods

Rats were anaesthetised with either 6% sodium pentobarbital (*n* = 14; Sigma‐Aldrich, Gillingham, UK) or isofluorane (*n* = 2; induced at 4% and maintained at 1.5–2%; Sigma–Aldrich) combined with oxygen (2 L/min). Chloramphenicol eye ointment (Martindale Pharmaceuticals, Romford, UK) was applied to their eyes to protect their corneas before the rats were placed in a stereotaxic frame (Kopf, Tujunga, CA, USA) with the mouth‐bar set at +5.0 mm. A sagittal incision was made along the scalp and a craniotomy made above the target site under aseptic conditions.

Multiple retrograde tracers were used to reduce the total number of animals as well as to compensate for the different labelling characteristics associated with the individual tracers and their visualisation (Craig *et al*., 1989). A 1‐μL Hamilton syringe (25‐gauge; Hamilton, Bonaduz, Switzerland) was used to inject 0.05 μL of 3% Diamidino Yellow (DY; Sigma–Aldrich), 0.03 μL of 3% Fast Blue (FB; Polysciences Inc., Eppelheim, Germany) diluted in phosphate‐buffered saline (PBS) (Bentivoglio *et al*., 1980) or 0.05 μL of a 0.5% solution (in PBS) of the non‐toxic B‐subunit of cholera toxin, Alexa‐Fluor 488 conjugate (CtB‐488; Life Technologies Ltd, Paisley, UK). Injections of 0.04 μL of 1% wheatgerm agglutinin (WGA; Vector Labs, Peterborough, UK) were made with a 0.5‐μL Hamilton syringe (25‐gauge). Horseradish peroxidase (HRP) conjugated with WGA (WGA‐HRP; Vector Labs) was used at a concentration of 40 mg/mL, with each injection consisting of 0.04–0.05 μL.

For the MBs, the injections clustered around the following co‐ordinates relative to bregma: AP, −2.1; medial–lateral (ML), ±0.8; and dorsal–ventral (DV), −10.4. Those injections that targeted the AV were placed at AP −0.4, ML ± 1.5 and DV −6.2 relative to bregma. Likewise, injections targeting the AM were placed at AP −0.2, ML ± 0.7 and DV −6.8 from bregma. The syringe was lowered to the target location and left in place for 3 min prior to injecting the tracer gradually over ~40 s, after which it was left in place for a further 7 min to help limit any tracer travelling back up the syringe tract. In all cases, a 5‐mL subcutaneous injection of 5% glucose in 0.9% saline was given after completion of surgery (Baxter Healthcare, Norfolk, UK). Aureomycin antibiotic powder (Fort Dodge Animal Health, Southampton, UK) was applied over the closed sutured scalp post‐surgery. Animals were then allowed to recover in a thermostatically controlled container before being returned to individual housing with *ad libitum* food and water. Following a post‐operative period, which varied depending on the tracer (WGA, 12–24 h; WGA‐HRP, 2 days; CtB‐488, FB and DY, 3–4 days), the animals were deeply anaesthetised with sodium pentobarbital (Euthatal; Merial, Harlow, UK). All rats were initially perfused transcardially with 0.1 m PBS at room temperature. In those animals that received fluorescent tracer injections or injections of WGA, the PBS was followed by 4% paraformaldehyde in 0.1 m PBS at 4 °C, while those animals that received injections of WGA‐HRP received a fixative consisting of 1.5% paraformaldehyde and 1.5–2% glutaraldehyde, again at ~4 °C.

**Figure 2:**
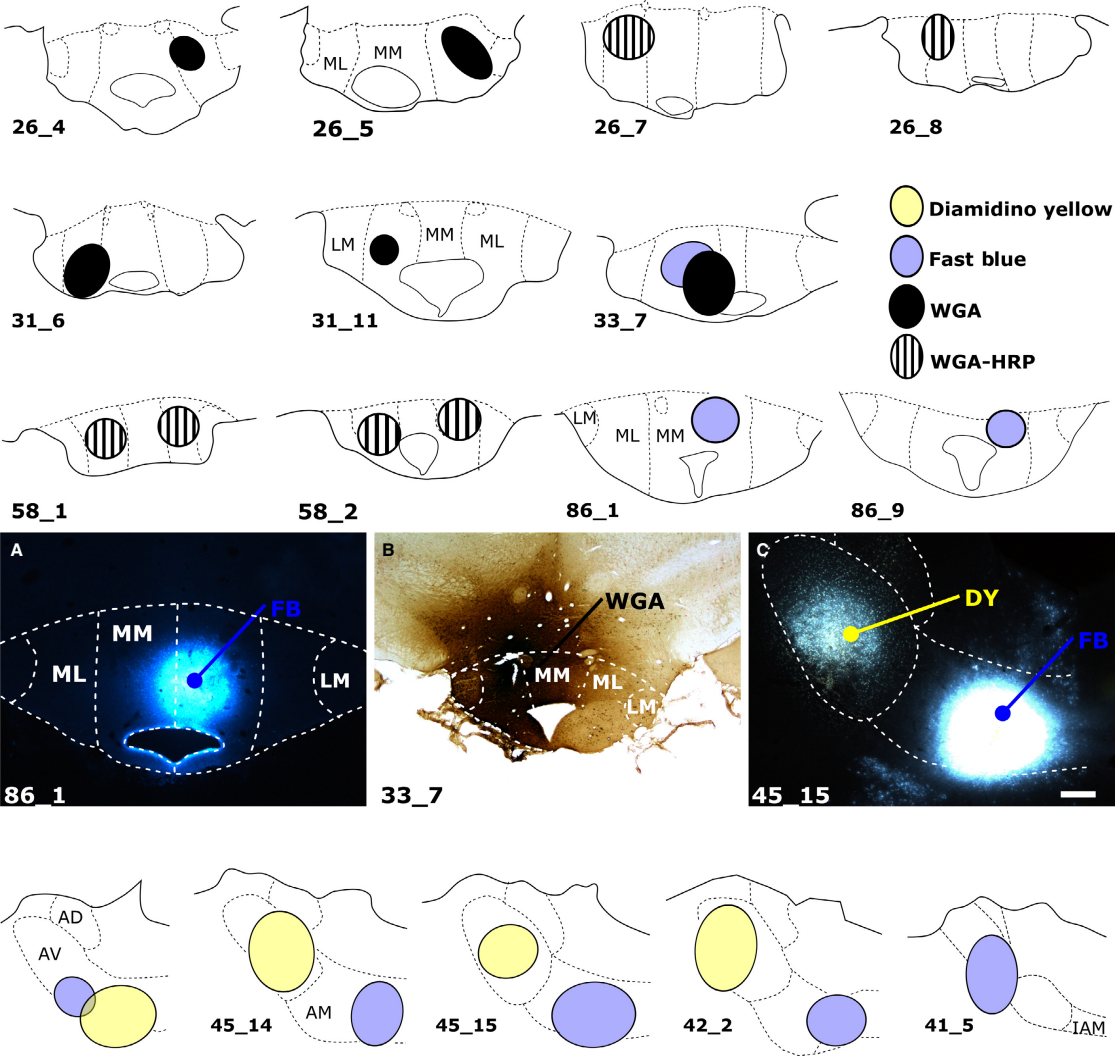
(Upper three rows and photomicrographs A and B) Coronal sections depicting the centre of each tracer injection in the rat MBs and (bottom row and photomicrograph C) in the rat anterior thalamic nuclei. Of the 11 cases with MB injections, some received unilateral injections of WGA (*n* = 4), WGA conjugated to HRP (WGA‐HRP,* n* = 2), or FB (FB,* n* = 2). The two FB injections (86_1 and 86_9) specifically targeted pars medialis (MM) of the medial mammillary nucleus (A), while the remaining cases were centred in pars lateralis (ML) of the medial mammillary nucleus (e.g. B). Of the three remaining cases, two received bilateral injections of WGA‐HRP into pars lateralis, while case 33_7 received injections of WGA and FB in pars lateralis of the same hemisphere. In the four anterior thalamic cases depicted (bottom row and C), the two tracers (DY and FB) were injected into either the AV or the AM. AD, anterodorsal thalamic nucleus; IAM, interanteromedial nucleus; LM, lateral mammillary nucleus. Scale bars, 250 μm.

**Figure 3:**
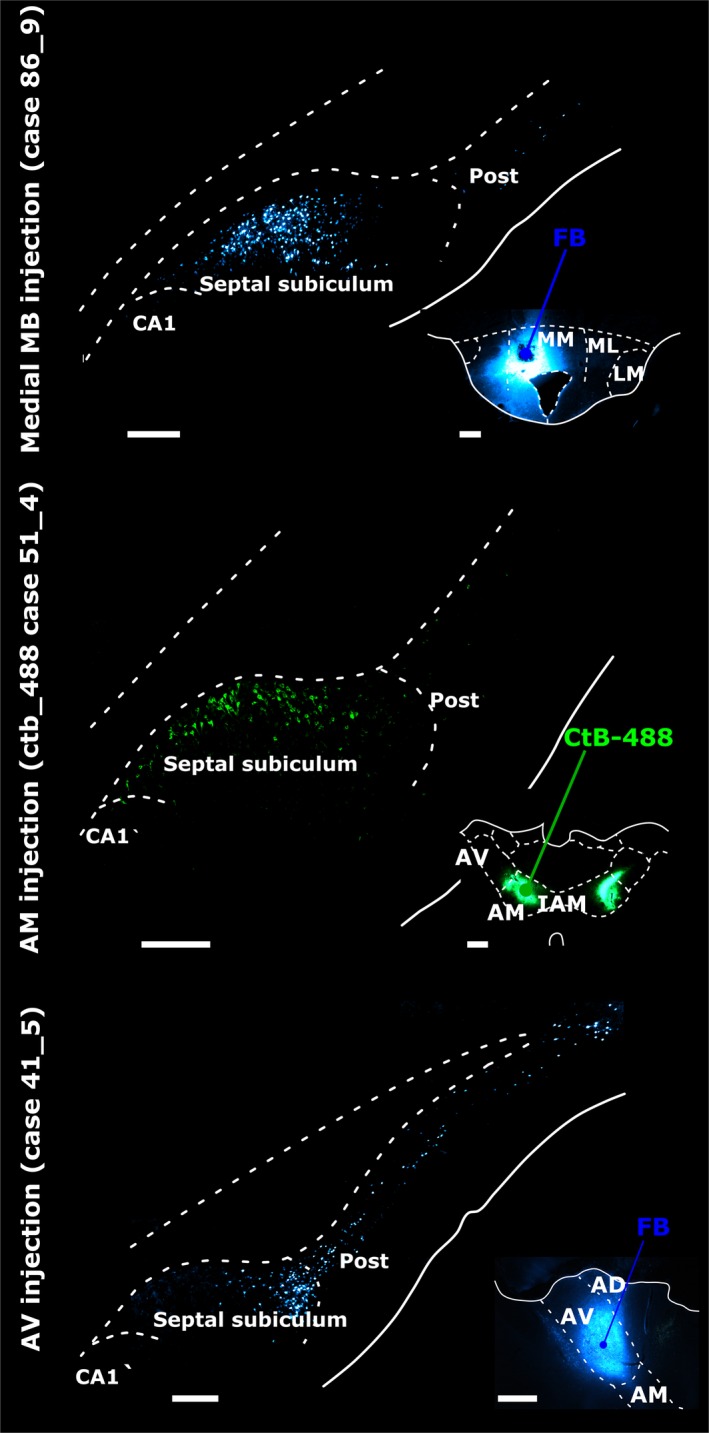
Retrograde neuronal label in the septal (dorsal) subiculum, postsubiculum (Post) and CA1 in the left hemisphere following tracer injections into (top panel) pars medialis of the medial mammillary nucleus (medial MB), (middle panel) the AM and (bottom panel) the AV. In each case, insets show injection site locations and tracer spread. Injections into the medial mammillary nucleus resulted in most labelled cells in mid‐subiculum regions R2 and R3 (top panel). Subiculum label density was greater proximal to CA1 following AM injections (middle panel), while tracer injections into AV resulted in most retrogradely label distal to CA1 (bottom panel). AD, anterodorsal nucleus; CA1, CA1 hippocampal subfield; CtB‐488, non‐toxic B‐subunit of cholera toxin, Alexa‐Fluor 488 conjugate; IAM, interanteromedial nucleus; LM, lateral MB nucleus; ML, pars lateralis of the medial mammillary nucleus; MM, pars medialis of the medial mammillary nucleus. Scale bars, 500 μm (injection sites), 250 μm (subiculum).

The eleven MB injection cases involved nine LH and two DA rats. Of these, four cases used just WGA while four just involved WGA‐HRP. Two other cases received unilateral injections of FB, while one case received both WGA and FB in the same hemisphere (Fig. 2). Of the six cases that received anterior thalamic injections, four received paired injections of FB and DY into the AV and AM, one case received a single FB injection into the AV and the final case received paired injections (one in each hemisphere) of CTB488 in the AM (Fig. 3).

#### Histology

Brains were removed from the skull and post‐fixed for 4 h in the fixative with which they were perfused, then transferred to a cryoprotectant solution containing 25% sucrose in 0.1 m PBS for 24 h. Brain sections were cut at 40 μm in the coronal plane using a Leica 1400 freezing microtome. For those cases with fluorescent tracers, two 1‐in‐3 series of sections were mounted directly onto gelatin‐subbed slides, before being dried in the dark at room temperature. For localising injection sites, one series was stained with Cresyl Violet while the second 1‐in‐3 series was rehydrated and cover‐slipped with either Hydromount (National Diagnostics UK, East Riding, UK) or DPX (Sigma–Aldrich, Gillingham, UK). For the cases with other tracers, a 1‐in‐3 series was stained with Cresyl Violet while the two remaining series were retained for immunohistochemistry.

#### Immunohistochemistry (NeuN and WGA)

NeuN and WGA (Vector Labs) were localised immunohistochemically (Horikawa & Powell, 1986). Initially, a 1‐in‐3 series of sections was washed (3 × 10 min) in 0.1 m PBS, followed by two further washes in 0.1 m PBST. Sections were then incubated for 48 h in a primary solution (anti‐WGA raised in goat; 1 : 2000 dilution, Vector Labs; anti‐NeuN; 1 : 5000, Chemicon, Chandlers Ford, UK) with 2% normal horse serum in 0.1 m PBST. Following incubation, sections were washed (3 × 10 min) in 0.1M PBST before being incubated for 2 h in a secondary antibody solution (for WGA, biotinylated rabbit antigoat at a 1 : 200 dilution; for NeuN, biotinylated horse antimouse at a 1 : 250 dilution; both from Vector Labs). After further washes in PBST (3 × 10 min), sections were incubated in the Vectastain ABC solution (Vector Labs) for 2 h, then washed in PBST twice for 10 min each followed by three further washes in 0.1M PBS. Sections were then reacted with diaminobenzidine (0.05% with 0.01%H_2_O_2_; DAB; Sigma–Aldrich). Once the desired level of staining was achieved, the reaction was stopped with washes in 0.1M PBS (3 × 10 min). Sections were then mounted, dehydrated and coverslipped, as above.

#### Histochemistry (WGA‐HRP)

Following tissue sectioning at 40 μm, one of the 1‐in‐3 series was mounted directly onto gelatin‐subbed slides while a second 1‐in‐3 series was collected in 0.1 m PBS (pH 6.0) for the subsequent 3,30,5,50‐tetramethylbenzidine (TMB) reaction for visualisation of transported WGA‐HRP (Fujii & Kusama, 1984). For the TMB reaction, sections were incubated, with agitation, in a fresh 0.1 m phosphate buffer (PB; pH 6.0) solution before being incubated at room temperature for 30 min in a solution containing 0.25% ammonium molybdate in 0.1 m PB and 0.002% TMB, dissolved in 100% ethanol. After incubation, a 1% hydrogen peroxide solution in distilled water was added in three stages, at 30‐min intervals, until the final concentration of hydrogen peroxide was 0.3%. Sections were then incubated in the same solution overnight at 4 °C. The TMB reaction precipitate was stabilized through subsequent incubation of sections in a 5% ammonium molybdate solution in 0.1 m PB (pH 6.0) for 30 min (see Fujii & Kusama, 1984; Marfurt *et al*., 1988). Next, sections were washed in 0.1 m PB (pH 6.0) before being mounted on gelatin‐subbed slides and left to dry overnight at room temperature. The sections were then dehydrated, coverslipped and mounted, as above.

#### Imaging

A Leica DM5000B microscope with a Leica DFC310FX digital camera and Leica Application Suite image acquisition software were used for brightfield and fluorescence microscopy. Images were then montaged using Microsoft Image Composite Editor (ICE).

#### Cell counts for different components of the subiculum

Sections from three adult male LH rats were stained with the neuronal marker NeuN (Jongên‐Relo & Feldon, 2002) to help estimate relative neuronal counts in different proximal–distal sectors of the subiculum. The purpose was to ensure that differences in the numbers of subicular cells containing retrograde tracer did not merely reflect the presence of more neurons in that same area. The cell counting procedures were not designed to give precise absolute counts, for which stereology would be needed. Instead, they provided relative counts with which to compare levels of labelled cells within each subicular region of interest.

Prior to cell counting, the subiculum was divided along its proximal–distal axis into four equally sized columnar regions of interest (see Fig. 1). These four regions of interest (R1–4) created a template for cell counting. In the case of NeuN, both hemispheres were counted, providing a mean count per animal. Next, the results from all three NeuN cases were combined to create an overall mean.

For the retrograde tracers, the cell counts were confined to the hemisphere ipsilateral to the injection site. For the MB injections, eight coronal sections, 240 μm apart, from the dorsal hippocampus were examined per rat. The sections began from close to where the septal subiculum first appears and ended as the dorsal and ventral subiculum fuse to form the intermediate subiculum, i.e. just before the structure flexes at 90° (Strange *et al*., 2014). In the ventral (temporal) subiculum, a further five coronal sections were analysed per case, again 240 μm apart. The same cell counting procedures were used for the anterior thalamic injection cases except that only the dorsal hippocampus (eight coronal sections) was examined in each rat. Counts were not made in the temporal subiculum as very few cells originate here to innervate the anterior thalamic nuclei (Meibach & Siegel, 1977; Sikes *et al*., 1977; Swanson & Cowan, 1977; Wright *et al*., 2010, 2013).

For the fluorescent tracers (FB and DY), each labelled cell was marked by hand using an ImageJ Cell Counter Plugin (Schneider *et al*., 2012). For those markers visualised chemically (NeuN, WGA and WGA‐HRP), positively stained cells were counted using an automated method. (Fluorescent label was not counted automatically as the signal lacked the same degree of contrast between stained and unstained cells.) First, NeuN, WGA and WGA‐HRP tracer images were converted into 8‐bit greyscale images. The images were then manually thresholded to encompass all particles that can be considered stained and any conjoined cells were split using the watershed tool. The acceptable particle size was set in the range 30–120 μm^2^ to limit the inclusion of glial cells or artefacts. Stained cells were counted using imagej software (Schneider *et al*., 2012).

#### Statistical comparison of subiculum cell counts

The statistical comparisons only considered within‐subject analyses, which contrasted the cell counts in the four proximal–distal regions of interest (Fig. 1) or the various AP levels. The data for these statistical analyses came from the estimates of the proportion of labelled cells from the total neuron population (NeuN) in each subicular subregion. Comparisons were not made between cases as there are many individual factors that could affect the overall numbers of labelled cells in each animal, e.g. type and amount of tracer.

All statistical and graphical analyses were performed in r (version 3.0.0; freely available from https://www.r-project.org/). Mixed‐effects models were generated with a random effect controlling for counts from multiple sections within a given case. Region of interest (i.e. R1–4) along the proximal–distal axis was treated as a categorical variable while distance along the septal–intermediate plane was treated as a continuous variable. In the case of proximal–distal topographies, alpha was controlled for in subsequent pairwise comparisons by using a Tukey *post hoc* correction. Given that degrees of freedom cannot be accurately determined from mixed‐effects models (Bates *et al*., 2015), *P*‐values obtained for mixed‐effects regression analyses along the septal–intermediate axis are the product of likelihood ratio tests, that is, the isolation of the significance of a fixed effect based on a comparison of nested models.

### Experiment 2: macaque monkeys

The experiment used archival anatomical data involving either cynomolgus monkeys (*Macaca fascicularis*) or rhesus monkeys (*Macaca mulatta*). The tracer injections were made 10–30 years prior to their present analysis. Despite this interval, comparisons with drawings involving the hippocampus made shortly after the injections confirm that the overall distribution of label appears unchanged. In the case of the HRP injections into the MBs, the quantity of retrograde label appears similar to that originally mapped. For the various thalamic injections, fading of the tracer signal has occurred over time. For this reason, cell counts from the thalamic injection case will underestimate the true amount of label, while injection sites may appear to constrict. Given the small number of animals and the variation between cases, only descriptive statistics are given for the monkey cases. Even so, within‐subject counts, e.g. along the AP and proximal–distal axes should still provide informative comparisons between regions of interest.

#### Anatomical nomenclature

The hippocampal nomenclature is taken from Lorente de Nó (1934). The subicular region comprises (from proximal to distal) the prosubiculum, subiculum, presubiculum and parasubiculum (see also Saunders & Rosene, 1988; Ding, 2013). The prosubiculum, a transitional region immediately adjacent to field CA1 (Lorente de Nó, 1934), is regarded as part of the subiculum, making it the most proximal region of interest. The distal limit of the subiculum is marked by the presubiculum, although some distal subicular cells often underlie the layer II cells of the most proximal presubiculum. Within the monkey subiculum, three distinct laminae can often be recognised: a superficial molecular layer, a pyramidal cell layer and a deep layer of polymorphic cells (Lorente de Nó, 1934).

The MB descriptions follow those of Veazey *et al*. (1982) for the cynomolgus monkey (see also Rose, 1939). For this reason, the medial mammillary nucleus can be subdivided into a medial, basal and lateral division. The thalamic borders are based on descriptions of the rhesus monkey thalamus by Olszewski (1952). As a consequence, three major anterior thalamic nuclei are recognised: AM, AV and anterodorsal.

#### Subjects

The tracer data came from eight adult monkeys (six cynomolgus and two rhesus monkeys). All experimental procedures were in strict adherence to the NIH Guide for Care and Use of Laboratory Animals, specifically the ‘Principles of Laboratory Animal Care’ (NIH Publication No. 86‐23, revised 1985). An additional adult rhesus monkey provided coronal sections for NeuN analysis.

#### Surgical method

##### MB injections

Subjects were three male cynomolgus monkeys (MB1–3) ranging in weight from 4.0 to 5.6 kg at the time of surgery. In all three cases a 0.15‐μL injection of 35% HRP (Boehringer, Mannheim, Germany) in 2% dimethyl‐sulfoxide solution was placed in the region of the left MB. Full descriptions of the surgical procedures have been given elsewhere (Saunders *et al*., 2012). In brief, monkeys were sedated with ketamine hydrochloride (10–15 mg/kg, intramuscular injection), then given intravenous pentothal, before being placed in a stereotaxic apparatus. After removal of a bone flap, a 1‐μL Hamilton syringe (Bonaduz, Switzerland; 25‐gauge) was lowered into the mammillary region using co‐ordinates determined from X‐ray skull landmarks (Aggleton, 1985). After 48 h the monkeys were perfused intracardially with physiological saline followed by 1 L of a solution of 2.5% paraformaldehyde and 1.5% glutaraldehyde in PB (pH 7.2). The brains were blocked in the coronal plane and then cryoprotected in cold 30% sucrose in (0.1 m) PB solution for 3 days. Frozen sections (50 μm) were cut in the coronal plane and collected in PB.

The series of sections examined in this study was processed according to a modified Hanker–Yates technique (Hanker *et al*., 1977; Perry & Linden, 1982). The Hanker–Yates procedure was as follows: the brain sections were incubated in 700 mL of a 0.1 m sodium cacodylate buffer solution (pH 5.1) containing: cobalt chloride, 2.4 g; ammonium nickel sulphate, 1.6 g; catechol, 700 mg; and p‐phenylenediamine, 350 mg for 15 min and then washed in PB for 3–5 min. The sections were then transferred into a fresh solution of catechol, 700 mg; p‐phenylenediamine, 350 mg; and hydrogen peroxide (H_2_O_2_), 1 drop, and incubated for 15 min. Sections were mounted on glass slides, counter‐stained with Cresyl Violet and coverslipped. Cells labelled with HRP were charted and counted on coronal sections at 1‐mm intervals.

##### Anterior thalamic injections: HRP cases

Three adult cynomolgus monkeys (ACy1, ACy2, ACy26) weighing from 3.5 to 6.8 kg received a single injection of HRP into the anterior medial thalamus under visual guidance. The initial surgical procedures were identical to those for the MB injections. Next, dorsal bone and dural flaps exposed the midline. The wall of one hemisphere was then gently retracted and a 5‐ to 10‐mm portion of the corpus callosum and the underlying fornix were split longitudinally to expose the thalamic midline. The largest HRP injection (case ACy1) involved a single stereotaxic injection of 0.22 μL (40% HRP; Sigma, St Louis, MO, USA; type IV) delivered via a 1‐μL Hamilton syringe (25‐gauge). Case ACy2 received a single injection of 0.13 μL of 40% HRP, while in case ACy26 a stereotaxic injection of 0.8 μL of a 4% solution of HRP (Sigma, type IV), conjugated with WGA, was targeted at the anterior thalamic region. Following injection of the tracer, the dura and skin were sutured in anatomical layers, and antibiotics and analgesia were given according to NIH veterinary guidance (see Aggleton *et al*., 2014). Recovery was without incident. After 48 h the monkeys were anaesthetised and perfused, and the brain treated exactly as described for the MB injections except that the perfusion used a solution of 1% paraformaldehyde and 1.25% glutaraldehyde in 0.1 m PB (pH 7.2). A 1‐in‐5 series was then treated with tetramethyl benzidine according to the protocol of Hardy & Heimer (1977). Alternate sections were dehydrated, counterstained with thionine, and coverslipped, while the remaining sections were just dehydrated and coverslipped.

##### Anterior thalamic injections: fluorescent tracer (FB)

Additional information came from two adult rhesus monkeys with FB injections into the anterior thalamic nuclei (BRh3, 4.9 kg; BRh5, 4.7 kg). This information is used selectively as in both cases there are complications concerning the tracer injections.

In case BRh3, FB was injected into the left anterior thalamic nuclei at the same time as a surgical transection of the ventroamygdalofugal pathway in the same hemisphere (Saunders *et al*., 2005). It is most unlikely that this tract surgery would disrupt direct inputs from the subiculum to the anterior thalamus (see Aggleton *et al*., 1986; Saunders *et al*., 2005). In the same case (BRh3), FB was also injected into the right medial dorsal thalamic nucleus, i.e. in the opposite hemisphere. The subiculum does not project to the medial dorsal nucleus. Furthermore, the fornix was sectioned in that same hemisphere, so disconnecting hippocampal inputs (see Saunders *et al*., 2005).

In case BRh5, FB was injected into the caudal left anterior thalamus. Although a second FB injection was located in the right laterodorsal thalamic nucleus, i.e. in the contralateral hemisphere, the projections from the hippocampus (subiculum) to the lateral dorsal nucleus are almost exclusively ipsilateral (Aggleton *et al*., 1986). For the reasons just explained, the tracer in the left subiculum in both BRh3 and BRh5 should overwhelmingly reflect the ipsilateral anterior thalamic injection. Nevertheless, greater reliance is placed on those cases with HRP injections in the thalamus.

The initial surgical procedures followed those described for HRP except that surgical anaesthesia was maintained with isoflurane (1% to 4%, to effect). Under visual guidance, an injection of FB (Sigma; 3% suspension in distilled water) was made through a 5‐μL Hamilton syringe fitted with a 28‐gauge needle. Following the tracer injection (~1 μL), the dura and skin were sutured in anatomical layers and antibiotics and analgesia were given according to NIMH veterinary guidance (see Aggleton *et al*., 2014). In both cases recovery was without incident.

After a post‐operative period of between 5 and 10 days, the animals with FB injections were deeply anaesthetised with sodium pentobarbital. They were then perfused intracardially with saline followed by 4–6% paraformaldehyde in 0.1 m cacodylate buffer (pH 7.4). The brains were then removed and placed in a series of cryoprotectant solutions consisting of first 10% and then 20% glycerol in 0.1 m cacodylate buffer with 2% dimethylsulfoxide and 4–6% paraformaldehyde (pH 7.4, 4 °C). Four to six days after perfusion, the brains were rapidly frozen by immersion in isopentane at −75 °C and then cut at 40 μm in the coronal plane on a freezing microtome (Rosene *et al*., 1986). Three × 1‐in‐10 series of sections were mounted immediately onto gelatine‐subbed slides, dried, coverslipped and stored in the dark at 4 °C.

#### NeuN staining

Staining was conducted in a male adult rhesus monkey (10.2 kg) that had received tracer injections into the auditory cortex (B.H. Scott, P.A. Leccese, K.S. Saleem, Y. Kikuchi, M.P. Mullarkey, M. Makoto Fukushima, M. Mishkin & R.C. Saunders, unpublished data). Following a survival period of 14 days, the animal was deeply anaesthetised with pentobarbital and perfused transcardially with 0.5 L of saline, followed by 0.5 L of 1% paraformaldehyde and 8 L of 4% paraformaldehyde, both in 0.1 m PB (pH 7.4) at room temperature. The brain was then removed from the skull, cryoprotected through a series of glycerols (Rosene *et al*., 1986), blocked in the coronal plane and frozen in isopentane at −80 °C. Sections were cut in the coronal plane on a sliding microtome at a thickness of 40 μm.

For NeuN immunohistochemistry, a 1‐in 5 series was retained. The sections were rinsed three times in Tris buffer solution (TBS) and subsequently agitated for 60 min in a blocking solution composed of 10% normal rabbit serum with 0.2% Tween‐20 in TBS, before additional TBS rinses. The sections were then incubated for 24 h in mouse anti‐NeuN (MAB377, 1 : 500; Chemicon) at room temperature. The next day sections were repeat‐rinsed with TBS and then incubated with pooled highly cross‐adsorbed secondary antibodies (antimouse Alexa Fluor 568, 1 : 250; Life Technologies, Grand Island, NY, USA). Sections were then mounted on slides and coverslipped with PVA‐DABCO (Sigma).

#### Cell counts for different components of the subiculum

Coronal sections taken from along the length of the hippocampus were stained with the neuronal marker NeuN (Jongên‐Relo & Feldon, 2002). The sections analysed came from an adult male rhesus monkey. As in the rat, the goal was to estimate relative neuronal counts in different proximal–distal sectors of the subiculum. The procedure for dividing the subiculum into four equidistant proximal–distal regions and for counting the NeuN cells was the same as that described for rats, i.e. automatic. These sections were then matched to the tracer section at the most comparable AP level.

In all cases with tracer injections, cell counting was manual to address the variable fading of the signal (least in the three MB cases with HRP injections, but most in the three thalamic cases with HRP injections). For the three MB injection cases and the two fluorescent (FB) thalamic injections, a total of 12 or 13 sections per animal were imaged and counted. The proximal and distal boundaries of the subiculum (along with R1–4) were first marked on the image. Cell counts were confined to the hemisphere ipsilateral to the injection site. For the three cases with anterior thalamic HRP injections, the number of remaining coronal sections through the subiculum varied, from eight (ACy2) to 27 (ACy26). Each section was treated as separate except in case ACy26, where the cell counts from adjacent slides were combined and averaged (mean). Cell counting in these three thalamic HRP cases was conducted at a higher magnification than in the other cases, in response to the fading of the HRP‐positive reaction product.

## Results

### Experiment 1: rats

**Figure 4:**
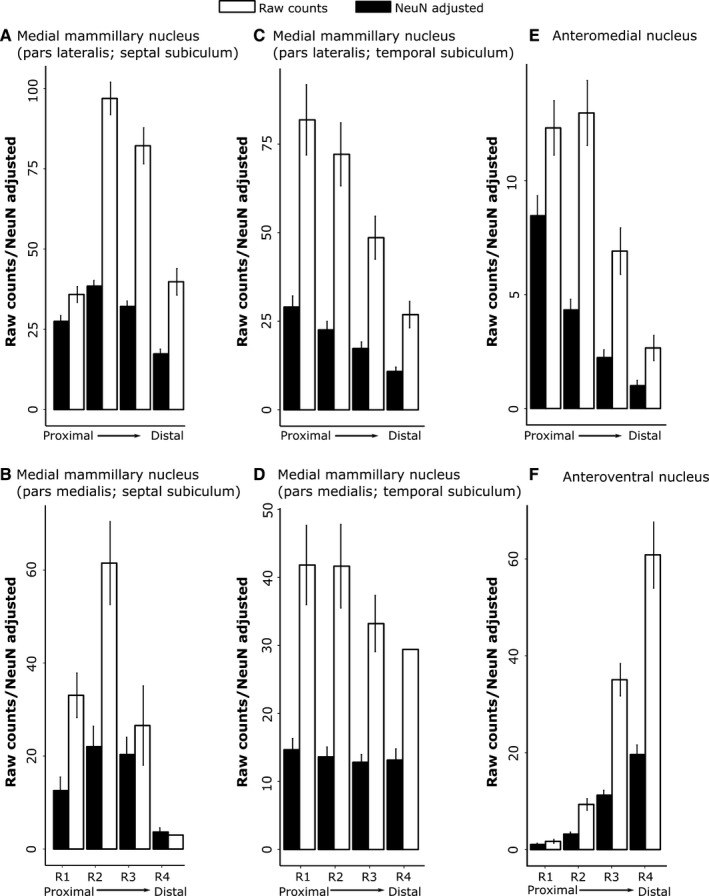
Rat subiculum: distribution of both the raw counts of retrogradely labelled cells (white) and the percentage of labelled cells from the total number (raw count) of NeuN cells within the same subicular region (‘NeuN adjusted’, black) along the proximal–distal axis. (A) Septal (dorsal) subiculum after injections into medial mammillary nucleus, pars lateralis; (B) septal (dorsal) subiculum after injections into medial mammillary nucleus, pars medialis; (C) temporal (ventral) subiculum after injections into medial mammillary nucleus, pars lateralis; (D) temporal (ventral) subiculum after injections into medial mammillary nucleus, pars medialis; (E) septal (dorsal) subiculum after injections into AM of the thalamus; and (F) septal (dorsal) subiculum after injections into the AV of the thalamus. Note that trends in raw cell count distributions are mirrored by those from adjusted cell counts. (The rat septal hippocampus is homologous to posterior primate hippocampus). The graphs show the mean scores ± 95% confidence intervals. (Note that the *x*‐axes have different scales, reflecting the different numbers of labelled cells.)

**Figure 5:**
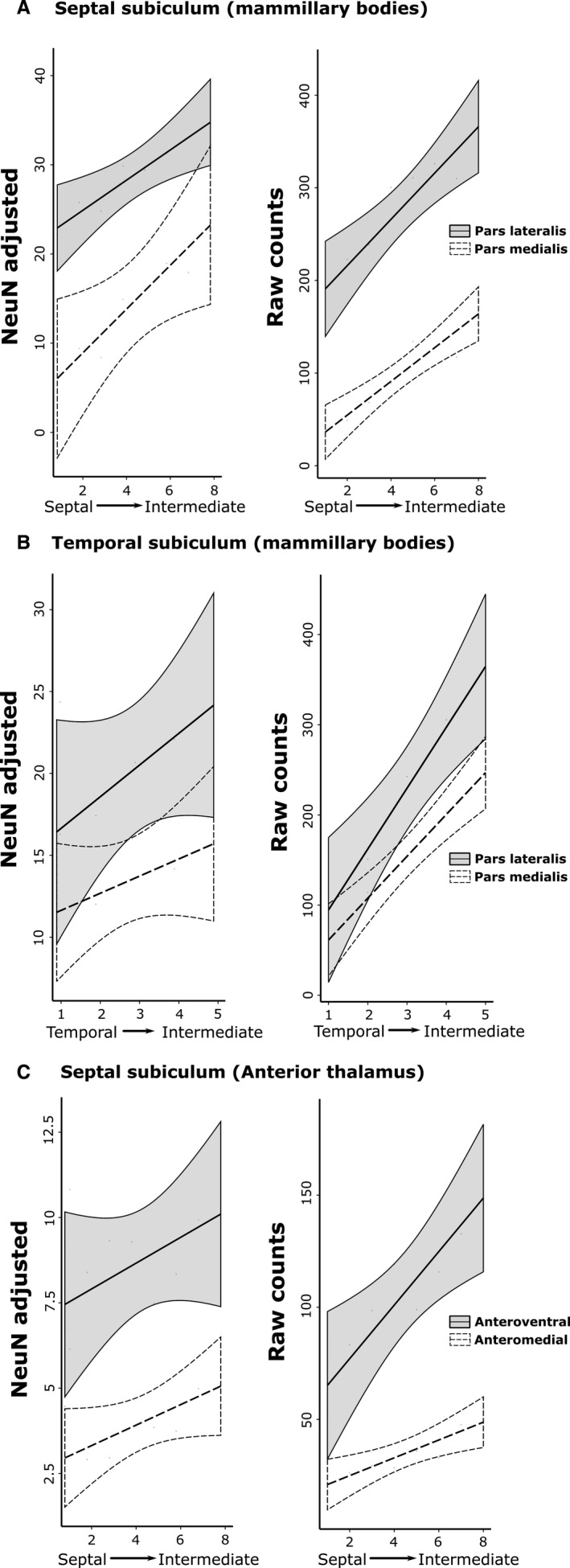
Rat subiculum: distribution of NeuN‐adjusted (left column) and raw (right column) cell counts in the AP plane. The graphs show the mean scores ± 95% confidence intervals. The *x*‐axis depicts successive slides in (A and C) the septal→intermediate hippocampal direction and (B) the temporal→intermediate hippocampal direction. (A) Results for the septal (dorsal) subiculum following tracer injections into the medial mammillary nucleus, pars lateralis and pars medialis. (B) Results for the temporal (ventral) subiculum following injections into the medial mammillary nucleus, pars lateralis and pars medialis. (C) Results for the septal (dorsal) subiculum following injections into the AV and AM. The NeuN‐adjusted scores are the percentage of labelled cells from the total number of NeuN cells in the corresponding region of interest. (Note that the *x*‐axes have different scales, reflecting the different numbers of labelled cells.)

Eleven cases, with a total of 14 injections, provided the quantitative data for the inputs to the MBs (Fig. 2). The five anterior thalamic injection cases used for cell counts were those in which the tracer injection was most clearly centred in either the AV or AM (Fig. 2). Cells projecting to the medial mammillary nucleus, the AV and the AM all displayed different topographies in the proximal–distal plane (Figs 1, 3 and 4). There were also clear differences in the AP plane when the findings from the MB injections were compared with those in the anterior thalamic nuclei (Fig. 5).

#### Subicular inputs to the MBs

The various retrograde tracer injections were centred in either pars lateralis (*n* = 9) or pars medialis (*n* = 2; FB, 86_1, 86_9) of the medial mammillary nucleus (Fig. 2). These injections consistently led to retrogradely labelled cells across the proximal–distal axis of the septal and adjacent intermediate subiculum (Figs 3, and 4A and B). The highest proportion of retrogradely labelled cells (relative to the NeuN counts) was, however, present on the proximal side of the central subiculum (i.e. R2; Figs 3 and 4). The lowest relative density of label was consistently in the distal most region (R4), which was significantly less than that in R1–3 (χ^*2*^ = 129.8, *P *<* *0.001; Fig. 4). At the same time, labelled cell density typically increased going from the septal to the intermediate hippocampus (1.82 ± 0.24, *t *=* *7.58, χ^2^ = 53.20, *P *<* *0.001; Fig. 5).

A subsequent comparison of these septal topographies involved separating those cases that received injections into pars lateralis or pars medialis (cases 86_1 and 86_9) of the medial mammillary nucleus. This comparison revealed that pars medialis injections resulted in a significantly reduced proportion of retrograde label in the distal‐most subiculum (i.e. R4; −14.20 ± 2.28, *t *=* *−6.22, *P *<* *0.001; Fig. 4A and B). Nevertheless, pars lateralis and pars medialis cases showed similar AP topographies, i.e. the proportion of labelled cells increased significantly from septal to intermediate hippocampus (pars medialis, 2.46 ± 0.74; pars lateralis, 1.69 ± 0.41; both *P *<* *0.001; Fig. 5).

Considerable retrograde label was also present in the temporal (ventral) subiculum following injections of tracer into either pars lateralis or pars medialis of the medial mammillary nucleus (Fig. 4C and D). Analyses of the topography of these ventral subicular inputs revealed a significant increase in the proportion of labelled cells going from temporal to intermediate levels (pars lateralis, 1.93 ± 0.73, *t *=* *2.66, χ^2^ = 6.67, *P *=* *0.01; pars medialis, 1.36 ± 0.63, χ^2^ = 4.17, *P *=* *0.04). Along the proximal–distal axis, the distribution of the proportion of retrogradely labelled cells following injections into pars lateralis decreased significantly (χ^2^ = 64.29, *P *<* *0.001) with more in R1 than the other regions R2–4 (R1 – R2, 6.42 ± 2.20, *t *=* *2.91, *P *=* *0.02; R1 – R3, 11.69 ± 2.20, *t *=* *5.31, *P *<* *0.001; R1 – R4, 18.47 ± 2.22, *t *=* *8.34, *P *<* *0.001). In contrast, no significant trend was observed in the proportion of labelled cell topographies along the proximal–distal axis following injections into pars medialis (all contrasts, *t *<* *1.04 and *P *>* *0.73).

#### Subicular inputs to the AM

Tracer injections in the AM consistently resulted in most labelled cells in the proximal subiculum and least in the distal subiculum (Fig. 4). Consequently, the highest proportion of labelled cells, relative to NeuN cell counts, was in the proximal subiculum, with the density decreasing significantly in the distal direction (Figs 1B, 3, and 4E; χ^*2*^ **= 104.73, *P *<* *0.001). The proportion of retrogradely labelled cells (NeuN‐adjusted) was found to increase significantly from septal to intermediate hippocampus (Fig. 5; 0.30 ± 0.09, *t *=* *3.44, χ^2^ = 11.34, *P *<* *0.001). In no case did the AM injection appear to cross the midline, although the injections typically spread medially to reach the edge of the interanteromedial nucleus. The lack of label in the temporal (ventral) subiculum meant that this region was not included in these analyses (also for the AV).

#### Subicular inputs to the AV

Injections into the AV revealed an increasing gradient of inputs from proximal (lowest) to distal (highest) subiculum (χ^2^ = 142.83, *P *<* *0.001; Fig. 4F). The proximal–distal gradient was, therefore, in the opposite direction to that found for inputs to the AM, i.e. a third distinct pattern. Alpha‐corrected pairwise comparisons for the AV inputs revealed a highly significant increase in NeuN‐adjusted cell density with each distal step (*t *>* *7.47, *P *<* *0.001), excluding the transition from R1 to R2 (2.15 ± 1.36, *t *=* *1.58, *P *=* *0.39). The proportion of retrogradely labelled cells was again found to increase significantly along the AP axis, albeit with greater variance than that described for the AM (0.38 ± 0.15, *t *=* *2.37, χ^2^ = 5.56, *P *=* *0.02).

### Experiment 2: macaque monkeys

The three cases with HRP injections into the medial MBs displayed extensive label across the mid, i.e. pyramidal, cell layer of the subiculum. In contrast, all of the monkeys with thalamic tracer injections had label confined within the deepest subiculum layer. For all diencephalic injections, the labelled cells were concentrated in the distal half of the subiculum. In fact, area R3 contained the highest number and highest proportion of labelled cells (relative to the total number of NeuN cells) in all cases, i.e. irrespective of whether the injection site was in the MBs or the anterior thalamus.

#### Subicular inputs to the MBs

**Figure 6:**
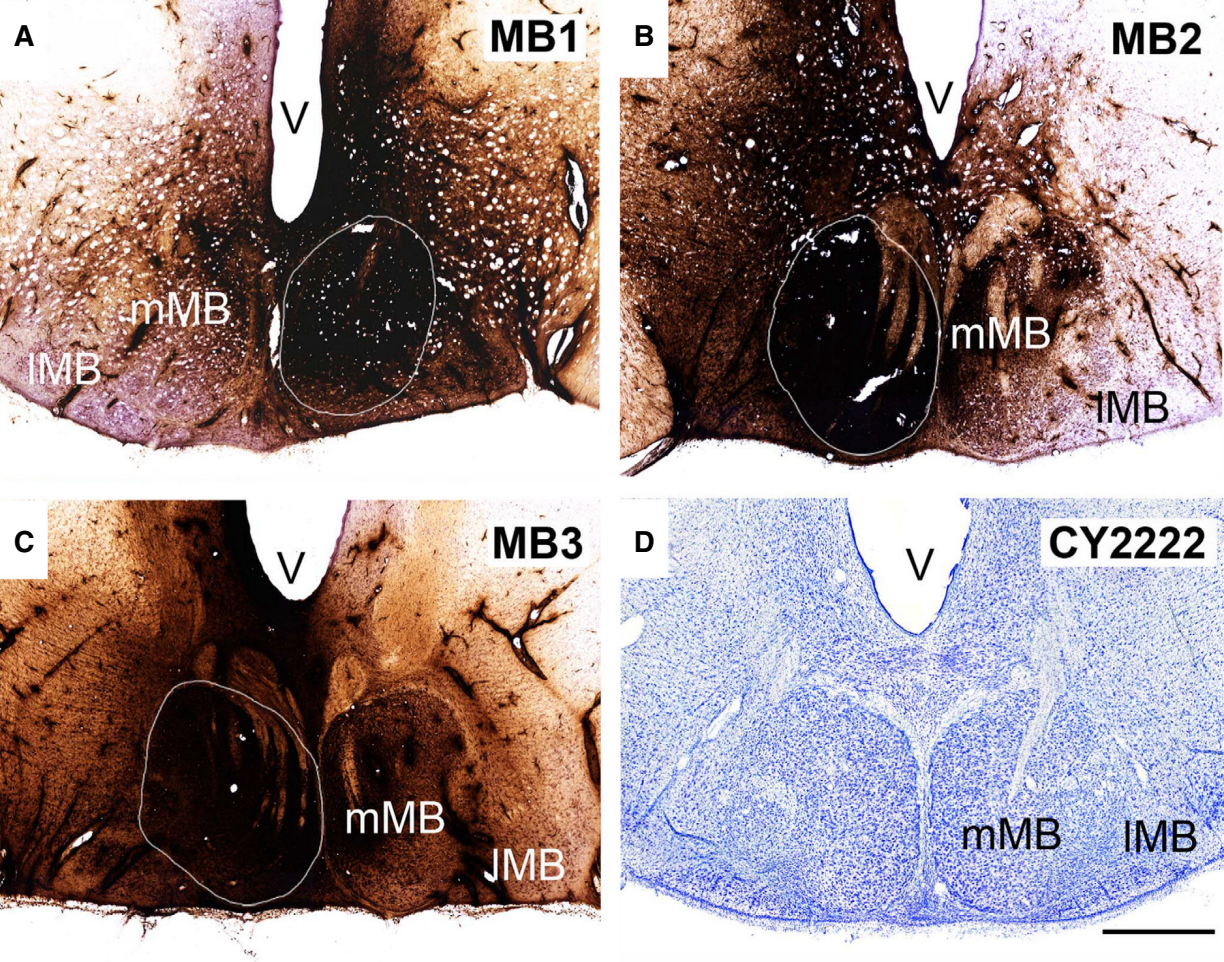
Neuronal tracer injection sites in the macaque MBs. (A–C) In three cases (MB1, MB2 and MB3, respectively), unilateral injections of HRP were made into the medial MB nucleus. (D) Cresyl Violet‐stained coronal section through the MBs of a cynomolgus monkey. lMB, lateral MB nucleus; mMB, medial MB nucleus; V, ventricle. Scale bar, 1 mm.

Three cynomolgus monkeys (MB1, MB2 and MB3) received HRP injections into the medial MBs (Fig. 6). The injection in MB3 was the most medial and that in MB2 was the most lateral. In all cases the injection appeared moderately large, such that reaction product extended laterally to reach the lateral mammillary nucleus, medially to the midline and seemingly beyond the nucleus. The active injection site was, however, almost certainly far smaller than the overall area of reaction product (see Saunders *et al*., 2012), and always centred in the medial mammillary nucleus.

The patterns of retrograde label across the proximal–distal subiculum were consistent across cases MB1–3 (Figs 8 and 9A). While considerable label was found across the proximal–distal plane of the subiculum, the highest proportion of labelled cells was repeatedly present in the mid proximal–distal subiculum (R2 and R3), with a consistent small bias towards the more distal parts of this region, i.e. R3 (Figs 8 and 9A). Only the most proximal subiculum (prosubiculum) contained light label. The subiculum label was confined to the pyramidal cell layer. In the AP plane, all three monkeys showed an obvious increase in labelled cells going from anterior to posterior subiculum (Fig. 9A). This increase was least marked in case MB3. A consistent feature in all three cases was that the laminar location of the label shifted when going from proximal to distal (Fig. 8). In the more proximal subiculum the cells projecting to the MBs were located deeply, i.e. just above the polymorphic layer, but they increasingly occupied a more superficial location when going more distal within the subiculum (Fig. 8).

#### Subicular inputs to the anterior thalamic nuclei (AM and AV)

**Figure 7:**
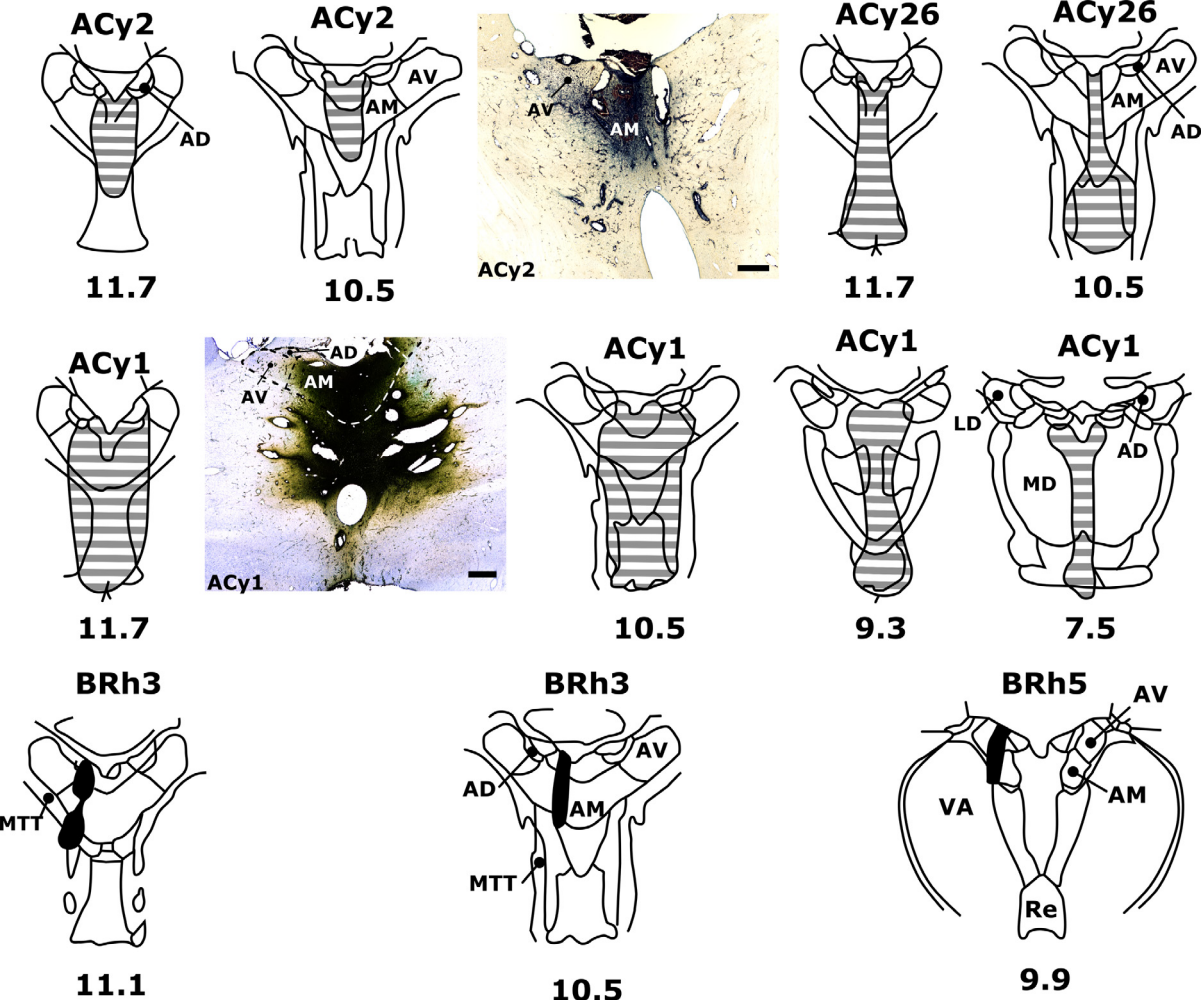
Neuronal tracer injection sites in the anterior thalamus of macaque monkeys. In four cases, injections of either HRP (cases ACy1, ACy2 and ACy26) or FB (case BRh3) were centred in the AM. In one additional case an injection of FB was centred in the caudal AV (case BRh5). Photomicrograph insets show tracer spread in coronal sections of the anterior thalamus for HRP cases ACy2 (centre) and ACy1 (mid left). Numbers below each schematic diagram represent AP levels, relative to bregma, according to Olszewski (1952). AD, anterodorsal nucleus; LD, laterodorsal nucleus; MD, mediodorsal nucleus; MTT, mammillothalamic tract; Re, nucleus reuniens; VA, ventral anterior nucleus. Scale bar, 500 μm.

Three cynomolgus cases had tracer injections that included the AM (Fig. 7). In cases ACy1, ACy2 and ACy26, single HRP injections involved the AM, with variable encroachment into adjacent nuclei. The largest injection of HRP (case ACy1) was centred in the AM but spread laterally to reach the border with the AV, caudally to reach the rostral medial dorsal nucleus and medial–ventrally to involve midline nuclei, potentially including nucleus reuniens (Fig. 7). In a second HRP case (ACy2) the injection was largely localised within the AM but again reached the midline (Fig. 7). The HRP injection in case ACy26 passed through the AM but extended ventrally to include nucleus reuniens (Fig. 7).

**Figure 8:**
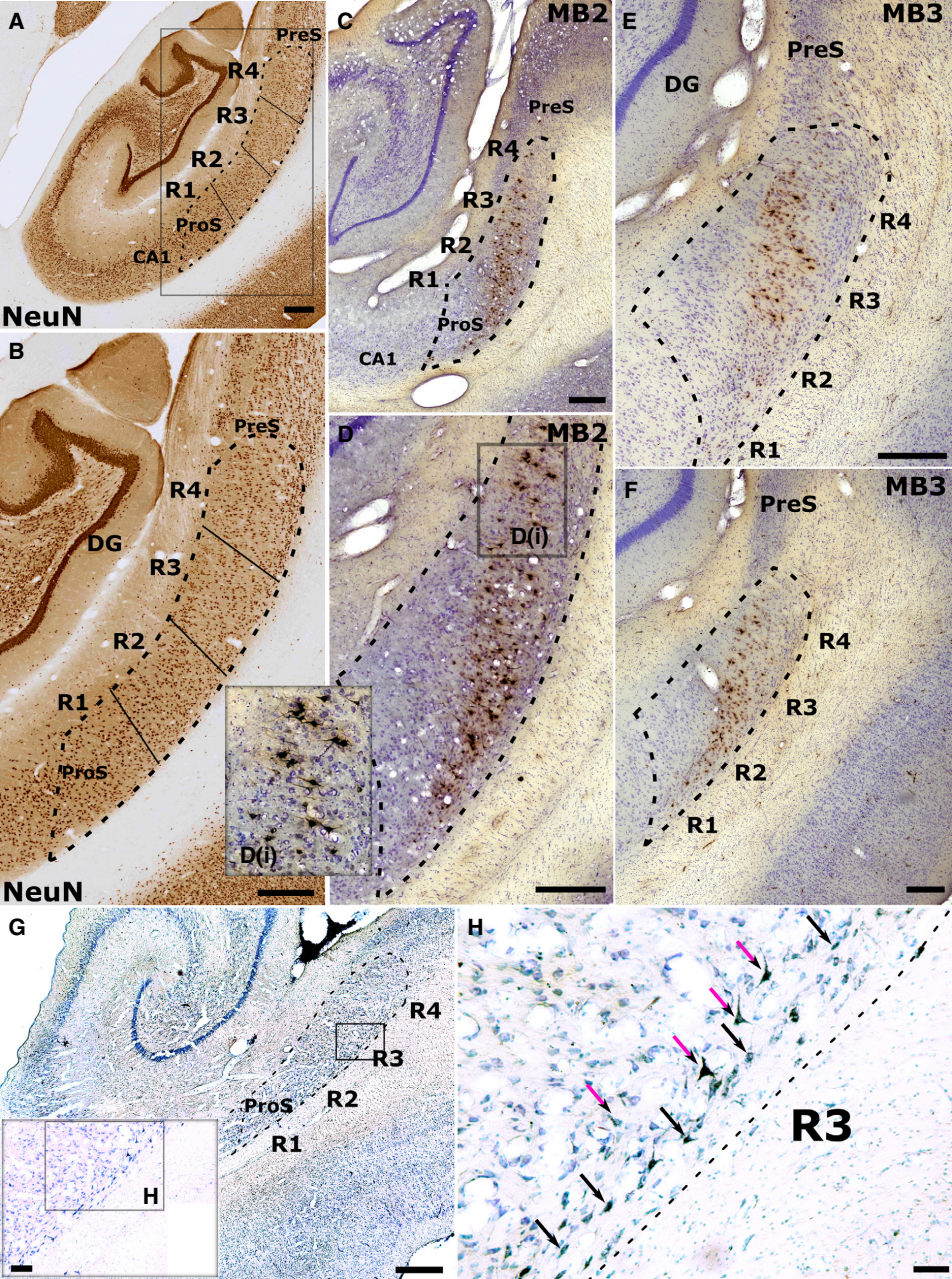
Retrograde label in the cynomolgus monkey subicular cortices following injections of HRP into either (C–F) the left medial mammillary nucleus (cases MB2 and MB3) or (G–H) the AM. (A and B) Low (A) and higher (B) magnification photomicrographs of a NeuN‐stained coronal section through the rhesus monkey hippocampus. Dashed lines indicate the boundaries of the subiculum while solid lines depict the boundaries of the four subiculum regions of interest (R1–4). R1 corresponds to the prosubiculum (ProS; most proximal to CA1) while R4 is most distal to CA1, i.e. next to the presubiculum (PreS). Following injections into the medial mammillary nucleus (MB2, MB3), retrograde label was concentrated in the more superficial pyramidal cells of R3 and R4 (distal subiculum), tapering off into deeper pyramidal cells when going more proximal (C–F). Inset D(i) shows retrograde label in more superficial R4 at higher magnification. G–H, low (G) and high (H) magnification photomicrographs showing retrograde label in the deep layer of the subiculum following an injection of HRP into AM (case ACy1). In ACy1 the label is in deep polymorphic cells (black arrows) and pyramidal‐like cells (purple arrows) nearer the upper border of this deep layer. (Note, sections G and H have been left–right reversed to aid comparison with the other cases.) The black rectangle and inset in G shows the region of high magnification shown in H. DG, dentate gyrus. Scale bars, 500 μm (A–G), 100 μm (H).

**Figure 9:**
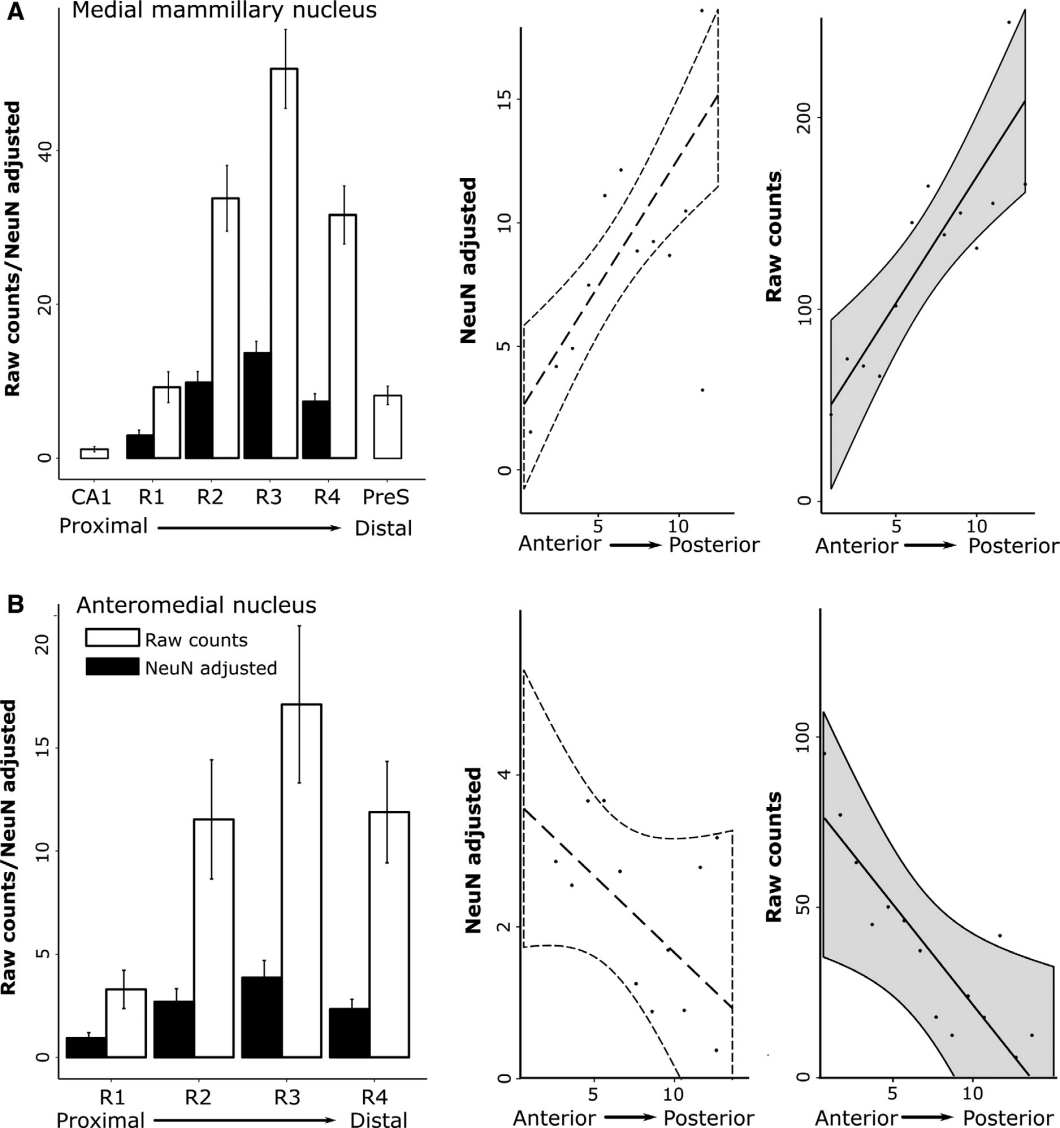
Distribution of retrogradely labelled cells in the macaque subiculum. (Left column) Distribution of the raw number of retrogradely labelled cells (white) and the percentage of these cells from the total number of NeuN cells (‘NeuN‐adjusted’; black) along the proximal–distal axis (R1–4). (Right column) Distribution of NeuN‐adjusted cell counts and raw cell counts (grey) along the AP hippocampal axis (*x*‐axis, sections 1–14). Cell distribution following HRP injections into (A) the MBs and (B) the AM. The graphs show the mean scores ± 95% confidence intervals. CA1, hippocampal field CA1; PreS, presubiculum.

Despite much variation in the total numbers of labelled cells across the three HRP cases, there was a consistent pattern in the distribution of subicular label. All three cases showed evidence of an AP gradient, with the anterior subiculum having the largest number of inputs to the AM (Fig. 9B). In the proximal–distal plane, the least label was in the most proximal subiculum, i.e. the prosubiculum (R1), while the third most distal region again contained the most label (Fig. 9B). This proximal–distal distribution was maintained along the AP axis of the subiculum. Throughout, the labelled cells were confined to the deepest cell layer so that most of the label was found in polymorphic cells, though some was also found in pyramidal cells at the upper junction of this lamina (Fig. 8H).

A fourth case (BRh3, rhesus) had an FB tracer injection in the left AM, but also an FB injection in the right medial dorsal thalamic nucleus. The pattern of cell counts in BRh3 matched that found in the three HRP cases. Although the most anterior three sections could not be counted, due to a localised infarct, the numbers of labelled cells consistently decreased going posterior, so that the most anterior sections (back of the uncus) contained the highest number of labelled subicular cells in BRh3 (~90 cells per section) while the most posterior sections contained about half that number. As with the HRP cases, the prosubiculum (R1) contained the fewest labelled cells while most were consistently found in R3, i.e. more distal.

The final case (BRh5, rhesus) had an FB injection centred in the more medial and caudal parts of the AV (Fig. 7), with a contralateral FB injection in the lateral dorsal thalamic nucleus. This case is of interest as, once again, the fewest labelled cells were found in the most proximal subiculum (i.e. the prosubiculum) while the two distal sectors contained the largest numbers of cells projecting to the thalamus. In the AP plane, however, the pattern of cell counts was very different from that observed after the AM injections as there was a gradual, consistent increase in label density going towards the posterior hippocampus. In both BRh3 and BRh5, the labelled cells were restricted to the deepest cell layer.

#### Fibres of passage

A potential concern is that the tracers might be taken up by fibres of passage, such as the fornix, through which many of the injection tracts passed. The selective nature of the hippocampal label (not only confined within the subiculum but also within a specific lamina) helped to establish that had such fibre uptake occurred it would not have provided the pattern of label observed [see Saunders & Aggleton (2007) and Wyss *et al*. (1980) for descriptions of the topographic organisation of white matter within the fornix in the macaque monkey and rat, respectively].

## Discussion

While previous studies have described hippocampal efferents to the diencephalon in either rats (Meibach & Siegel, 1975, 1977; Swanson & Cowan, 1977; Naber & Witter, 1998; Ishizuka, 2001; Wright *et al*., 2010, 2013) or monkeys (Krayniak *et al*., 1979; Aggleton *et al*., 1986, 2005; Xiao & Barbas, 2002b), the present study quantified and compared, where possible, the pattern of these connections both within and across species. The data from the monkey experiments were limited by both the numbers of cases and variations in their methodology. For this reason, comparisons involving these cases are descriptive. A particular focus throughout the study concerned how the origins of these diencephalic projections respect different hippocampal planes.

**Figure 10:**
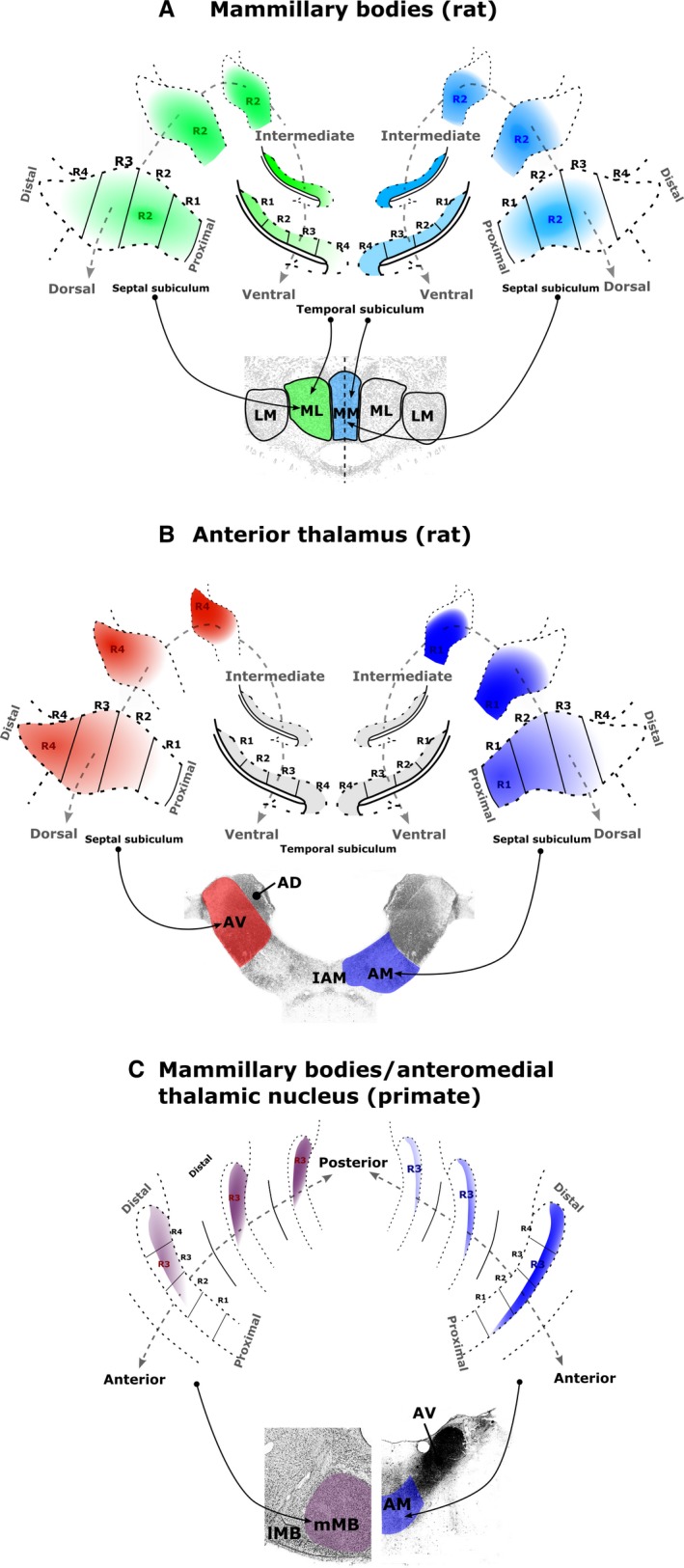
Schematic summaries of the topographic sources of the subicular inputs to (A) the rat medial MBs, (B) the rat anterior thalamic nuclei and (C) the macaque medial MBs (left) and AM (right). Denser sources of subicular label correspond to denser colours. (A) Subicular inputs to the rat medial mammillary pars lateralis (green) and pars medialis (blue) arise predominantly from regions R1–3. For both mammillary sub‐nuclei, the proportion of subiculum‐projecting cells increases approaching the intermediate hippocampus. (B) Inputs to the rat AV (red) arise predominantly from distal regions (R3–4) of the septal–intermediate subiculum, while the corresponding inputs to the rat AM (blue) mainly arise from the proximal regions (R1–2) of the septal–intermediate subiculum. (C) Subicular cells that project to the macaque medial MBs (left) are more numerous in the posterior hippocampus while subicular cells that project to the AM are more numerous in the anterior hippocampus. The two sets of inputs predominantly arise from the distal subiculum (especially R3) but from different laminae. AD, anterodorsal thalamic nucleus; LM, lateral mammillary nucleus; ML, medial mammillary nucleus, pars lateralis; MM, medial mammillary nucleus, pars medialis.

Consistent with previous studies (Meibach & Siegel, 1975, 1977; Sikes *et al*., 1977; Aggleton *et al*., 1986, 2005; Naber & Witter, 1998; Kishi *et al*., 2000; Ishizuka, 2001; Wright *et al*., 2010, 2013), the outputs to the thalamus and MBs were clearly segregated by their laminae of origin in both rats and monkeys (see also Ishizuka, 2001; Aggleton *et al*., 2005). With respect to the AP axis, dense inputs to the rat MBs arose from along the length of the subiculum while those to the anterior thalamic nuclei were largely confined to the septal and intermediate hippocampus, i.e. the posterior hippocampus (see also Meibach & Siegel, 1975; Kishi *et al*., 2000; Wright *et al*., 2010). In the macaque, the majority of projections to the MBs came from the posterior subiculum, while the inputs to the AM predominantly arose from the anterior subiculum. Finally, in the proximal–distal plane there were opposing gradients of inputs to the rat AV and AM (Figs 4 and 10), with inputs to the rat medial mammillary nucleus showing a third pattern. The majority of MB projections from the septal hippocampus arose from the central subiculum while more proximal inputs arose from the temporal subiculum (Fig. 10). In contrast, the macaque subiculum showed similar profiles of label in the proximal–distal plane for all of the diencephalic targets examined, with the greatest number of projections consistently arising from the more distal (R3) subiculum (Fig. 10).

Comparisons among cell laminae showed how MB inputs arose from intermediate layers while the thalamic inputs arose from the deepest subiculum cell layer. In the rat, these thalamic projections arose from modified pyramidal cells, while in the monkey much of this input came from deep polymorphic cells (see also Aggleton *et al*., 1986, 2005; Xiao & Barbas, 2002b). A similar laminar segregation is also seen in the squirrel monkey (Krayniak *et al*., 1979), implying that this is a very general feature of subiculum organisation. In the rat it has been possible to further confirm this laminar separation as individual subiculum cells do not project to both the MBs and the anterior thalamus (Wright *et al*., 2010). Based on their parahippocampal projections, it has been proposed that the deepest subiculum layer in the rat (thalamic inputs) is comparable to isocortical layer VI, while more superficial pyramidal cells (mammillary inputs) reflect isocortical layer V (Honda & Ishizuka, 2015).

Additional information comes from a study that also placed retrograde tracers in the AM of macaque monkeys (Xiao & Barbas, 2002b). Again, the distal subiculum was depicted as the major source of hippocampal inputs, with projections to the AM present along the full AP axis of the hippocampus. Intriguingly, that same study described some additional anteromedial thalamic inputs from CA3 (Xiao & Barbas, 2002b), something not observed in the present material.

The remainder of the discussion will consider the implications of the various topographies, first with respect to the AP and then the proximal–distal plane of the hippocampus (see Fig. 10).

### AP plane

Research with rodents has repeatedly pointed to a greater specialisation in the septal (i.e. posterior) hippocampus for processing high‐resolution spatial information (Moser *et al*., 1995; Bannerman *et al*., 1999; Fanselow & Dong, 2010). One general conceptualisation, based initially on electrophysiological findings from rodent studies, is that the anterior hippocampus provides coarse, global representations while the more posterior hippocampus is required for fine‐grained local representations (Poppenck *et al*., 2013; Collin *et al*., 2015). Other models also emphasise spatial information processing in the posterior hippocampus but with a transition to more emotion‐related responses, including stress and anxiety, in the anterior hippocampus (O'Mara, 2005; Strange *et al*., 2014; Chase *et al*., 2015).

These functional models can readily be linked to the projections from the rat subiculum. The greater concentration of MB and anterior thalamic inputs from the posterior half of the rat hippocampus, which is most marked for the anterior thalamic projections, fits with the considerable amount of evidence highlighting the importance of these same diencephalic sites for spatial learning (Beracochea & Jaffard, 1987; Sutherland & Rodriguez, 1989; Vann & Aggleton, 2003; Vann, 2005, 2010; Aggleton & Nelson, 2015). Furthermore, disconnection evidence confirms that the rat anterior thalamic nuclei function conjointly with the hippocampus to support spatial learning (Warburton *et al*., 2001; Henry *et al*., 2004), with both the AV and AM involved (Aggleton *et al*., 1996; Byatt & Dalrymple‐Alford, 1996; Aggleton & Nelson, 2015). In the case of the rat MBs, hippocampal inputs also arise from the temporal (‘anterior’) subiculum, where there is a slight change in the proximal–distal profile of subiculum cells. These temporal subiculum projections terminate in the ventral MBs while the septal (i.e. ‘posterior’) subiculum preferentially terminates in the dorsal MBs (Meibach & Siegel, 1975, 1977; Kishi *et al*., 2000), implying functional differences within the medial mammillary nucleus (see also Shibata, 1992; Hopkins, 2005).

There is growing evidence that human memory processes follow similar hippocampal gradients, such that increasingly refined contextual processing in the posterior hippocampus enhances the details of episodic memory (Woollett *et al*., 2009; Poppenck & Moscovitch, 2011; Ranganath & Ritchey, 2012; Poppenck *et al*., 2013; Strange *et al*., 2014; Collin *et al*., 2015). This functional gradient would appear to match the monkey medial MB inputs, which show a gradual AP shift, with most projections arising from the posterior hippocampus. As with rats, the macaque MBs are linked with scene and spatial learning (Aggleton & Mishkin, 1985; Parker & Gaffan, 1997), consistent with human structural and functional imaging findings that point to a relative posterior hippocampal specialisation for space and navigation (Woollett *et al*., 2009; Poppenck *et al*., 2013; Strange *et al*., 2014; see also Ranganath & Ritchey, 2012). There is also considerable clinical evidence highlighting the importance of the human MBs for episodic memory (Dusoir *et al*., 1990; Vann & Aggleton, 2004; Tsivilis *et al*., 2008; Vann, 2010; Vann & Nelson, 2015). It is, therefore, intriguing that the posterior hippocampus is more closely associated with accurate episodic memory (Poppenck & Moscovitch, 2011; Poppenck *et al*., 2013; Strange *et al*., 2014; Collin *et al*., 2015), thus matching these mammillary gradients. In the light of their shared roles in spatial navigation and episodic memory, it is also notable that the monkey retrosplenial cortex receives inputs from subicular cells that share the same topography in all three planes with the cells that project to the MBs (Kobayashi & Amaral, 2003; Aggleton, 2012). The potential significance of these shared features stems from the importance of the human retrosplenial cortex for aspects of episodic memory (Maguire, 2001; Epstein, 2008; Vann *et al*., 2009; Auger *et al*., 2012), i.e. functions that seemingly overlap with those of the MBs.

The projections to the primate AM predominantly arose from the anterior subiculum, a gradient shared with some other hippocampal efferents, e.g. those to the prefrontal cortex, perirhinal cortex, the amygdala and nucleus accumbens (Carmichael & Price, 1995; Aggleton, 1986; Saunders & Rosene, 1988; Barbas & Blatt, 1995; Blatt & Rosene, 1998; Friedman *et al*., 2002). The inclusion of prefrontal cortex is intriguing as it is the AM, rather than the AV, that is particularly interconnected with prefrontal cortex (Kievet & Kuypers, 1977; Xiao & Barbas, 2002a,b). The implication is that the AM provides an indirect route that interlinks the rostral subiculum with prefrontal cortex, alongside the direct prefrontal connections from the subiculum (Rosene & Van Hoesen, 1977; Carmichael & Price, 1995; Xiao & Barbas, 2002a; Aggleton *et al*., 2015). A further prefrontal route is probably provided by nucleus reuniens, which also receives direct inputs from the subiculum (Aggleton *et al*., 1986). Indeed, two of the HRP injections (ACy1, ACy26; Fig. 7) appeared to reach nucleus reuniens, although the distribution of subiculum label appeared no different from the two AM injections cases in which this midline nucleus was not involved. This apparent lack of difference might reflect the lightness of the projections from the subiculum to nucleus reuniens in macaque monkeys (Aggleton *et al*., 1986).

Our preliminary evidence (case BRh5) also suggests that some subicular–thalamic inputs in the primate brain (potentially to the AV) have the opposite AP gradient, i.e. they principally arise from the posterior hippocampus. This caudal hippocampal gradient, also shown by MB outputs, is shared with connections to the retrosplenial cortex, caudolateral entorhinal cortices, anterior cingulate cortex (area 24) and area TE (Suzuki & Amaral, 1994; Yukie, 2000; Insausti & Munoz, 2001; Kobayashi & Amaral, 2003; Aggleton *et al*., 2005). It has been suggested that the AV in rodents provides a ‘return loop’, taking information back to the hippocampal formation, in contrast to the ‘feed‐forward’ prefrontal connections of the AM (Aggleton *et al*., 2010; see also Xiao & Barbas, 2002a). Unfortunately, the return connections in the primate brain from the thalamus to the hippocampus are poorly understood. While it is known that all three anterior thalamic nuclei, as well as the laterodorsal nucleus, project directly to the hippocampal formation of monkeys (Amaral & Cowan, 1980; DeVito, 1980), the density of these inputs, including the balance between the AM and AV, along with their sites of termination within the hippocampus, remain to be detailed. It is also known that the thalamus projects directly to the entorhinal cortex, though these inputs arise from midline nuclei rather than the anterior thalamic nuclei (Insausti *et al*., 1987). Clearly, there is a need to improve our understanding of thalamohippocampal inputs in the primate.

### Proximal–distal axis

The proximal–distal gradients are first considered in the context of other connections that share similar topographies (Aggleton & Christiansen, 2015). In the rat, the proximal subiculum projections to the AM overlap with the sources of inputs to the lateral entorhinal cortex, perirhinal cortex and prelimbic cortex (Jay & Witter, 1991; Naber & Witter, 1998; Naber *et al*., 2000; Witter *et al*., 2000; Ishizuka, 2001; Kloosterman *et al*., 2003; Aggleton, 2012). Unlike the anteromedial thalamic connections, these hippocampal–cortical projections also originate from distal CA1. Based on these interactions, e.g. with the perirhinal cortex and lateral entorhinal cortex, the rat proximal subiculum might be expected to preferentially process object‐based information (Witter *et al*., 2000; Bussey & Saksida, 2007; Diana *et al*., 2007; Ahn & Lee, 2015). In contrast, the rat distal subiculum is more closely connected with the medial entorhinal cortex and postrhinal cortex, regions containing positional and navigational information (Burwell & Hafeman, 2003; Fyhn *et al*., 2004; Hafting *et al*., 2005).

The evidence for a functional proximal–distal gradient in the rat remains, at present, preliminary, with most support coming from electrophysiological studies. The two principal cell types in the rat subiculum are ‘bursting’ and ‘spiking’, names that reflect their electrophysiological properties (O'Mara, 2005). While the proximal subiculum consists mainly of spiking neuronal cell types (~80%), the distal subiculum consists mainly of bursting neuronal cell types (~80%; Kim & Spruston, 2012). As might be predicted from the present results, the hippocampal projections to the rat anterior thalamus comprise a mixture of the two cell types (Kim & Spruston, 2012).

Cells with spatial firing properties are found in the subiculum. Place firing by subiculum cells shows more coherence in the distal subiculum, associated with higher firing rates than the proximal subiculum (Sharp & Green, 1994). Further comparisons indicate that spatial firing in the distal subiculum has a higher information content than the proximal subiculum (Kim *et al*., 2012). More indirect evidence comes from the finding that proximal CA1 activity (most closely interlinked with distal subiculum) has greater spatial resolution than distal CA1 (most closely interlinked with proximal subiculum; Henriksen *et al*., 2010). Preliminary evidence for the complementary gradient (object‐based processing in the proximal subiculum) comes from the differential activity of distal CA1 cells, as measured by *Arc* expression, for nonspatial learning (Nakamura *et al*., 2013; see also Hunsaker *et al*., 2008). Caution is, however, required as some electrophysiological studies of subicular spatial cells, e.g. boundary vector cells and place cells, have failed to find proximal–distal differences (Lever *et al*., 2009; Brotons‐Mas *et al*., 2010).

Anatomical proximal–distal topographies exist in the monkey hippocampus, with the proximal subiculum (and distal CA1) containing the majority of projections to prefrontal cortex, amygdala and nucleus accumbens, as well as connections with the perirhinal and rostral entorhinal cortices (Van Hoesen *et al*., 1979; Aggleton, 1986; Saunders & Rosene, 1988; Suzuki & Amaral, 1990; Witter & Amaral, 1991; Blatt & Rosene, 1998; Insausti & Munoz, 2001). In contrast, the distal subiculum is preferentially interconnected with both caudal and lateral entorhinal cortex, as well as the parahippocampal areas TH and TF (Aggleton, 2012). These connections would again seem to provide a potential distinction between object‐based (proximal) and scene‐ or context‐based (distal) connections within the monkey subiculum (Diana *et al*., 2007; Murray *et al*., 2007; Ritchey *et al*., 2015). Unlike rats, however, the majority of inputs to the medial diencephalon consistently arose from the distal half of the monkey subiculum, suggesting a bias towards scene‐ or context‐based information.

An aim of the present study was to help determine whether the Papez circuit is organised so that it could provide high‐resolution, parallel information streams from the hippocampus to the medial diencephalon (Aggleton *et al*., 2010; Jankowski *et al*., 2015; Vann & Nelson, 2015). There is, for example, a potential distinction between object‐based (proximal subiculum) and context‐based (distal subiculum) information streams (Aggleton, 2012; Nakamura *et al*., 2013; Ritchey *et al*., 2015), seemingly reflected in the respective connections of the rat subiculum with the anteromedial and anteroventral thalamus. In contrast, the sources of the inputs to the rat MBs are more widely distributed across both the proximal–distal plane and the AP plane of the hippocampus. Given that the MBs then project very densely, in a topographic manner, upon the anterior thalamic nuclei (Shibata, 1992; Hopkins, 2005), there remains the potential for object‐based and context‐based information to be relayed indirectly to different parts of the anterior thalamic nuclei. A further segregation arises from the laminae separation of the subicular inputs to the MBs and the anterior thalamic nuclei, raising the question of whether the cell populations in this plane of the subiculum process different information types (Naber & Witter, 1998; Wright *et al*., 2013). For the monkey, a further challenge is to understand why the same proximal–distal zone (R3) contains the highest proportion of inputs to these various medial diencephalic targets. One intriguing clue comes from functional imaging evidence that the distal subiculum, along with presubiculum and parasubiculum, may be especially involved in the mental construction of scenes (Zeidman *et al*., 2015).
